# Tailoring Statewide Training for Local Impact: A Burke County Public Health Case Study

**DOI:** 10.13023/jah.0701.07

**Published:** 2025-05-01

**Authors:** Miranda Smith, Ashley Jarrett, Danny Scalise, Samantha Simmons

**Affiliations:** Burke County Public Health; Burke County Public Health; Burke County Health Department; Burke County Public Health

**Keywords:** Appalachia, Burke County, NCCARE360, North Carolina, Public Health, Tailoring, Training, Referral, Intervention

## Abstract

Addressing health-related social needs is crucial for improving health outcomes, especially for vulnerable populations. NCCARE360 is North Carolina’s statewide coordinated care platform powered by Unite Us.^1^ Burke County Public Health (BCPH) identified gaps in the standardized NCCARE360 training, prompting a tailored approach. Using the Plan-Do-Study-Act cycle, BCPH refined training materials with interactive guides, real-life scenarios, and one-on-one sessions. This adaptation led to increased staff engagement and improved referral outcomes. Recognized as a model for local implementation, BCPH’s approach signifies the importance of customizing statewide initiatives to meet local operational realities. This case highlights the value of iterative training improvements for optimizing public health interventions in resource-limited settings. Effective use of NCCARE360 will enhance referrals to essential services, helping to address barriers caused by the social determinants of health often seen in the larger Appalachian Region.^2^

## INTRODUCTION

NCCARE360, North Carolina’s statewide coordinated care platform powered by Unite Us, connects individuals with essential community resources addressing social determinants of health, such as food, housing, and employment.^1^ It streamlines service coordination among healthcare, government, and nonprofit organizations, with free access for community-based and safety-net providers. Burke County Public Health (BCPH) adopted NCCARE360 to advance its strategic vision of improving community health and reducing disparities.^3^ Despite Burke County’s rich Appalachian heritage, economic challenges persist, limiting job and educational opportunities.^4^ Public health systems face ongoing funding and staffing shortages, prompting workforce initiatives like the Public Health Workforce Calculator and Staffing Up.^5^,^6^ However, BCPH found that state-provided NCCARE360 training materials did not align with its unique operational needs, leading to refined onboarding strategies for better integration into clinical workflows. By examining the effects of implementing this new approach to training, NCCARE360’s growth and development may provide a source of perspective for the betterment of broader Appalachian health administration.

## IMPLEMENTATION OF TAILORED TRAINING

In 2024, BCPH launched a pilot integration of NCCARE360 into clinical workflows for internal programmatic referrals. Data was collected for one year. To refine the training process, the department employed principles of active implementation and the Plan-Do-Study-Act cycle,^7^ a structured framework for testing and refining programmatic changes. The goal was to identify gaps in the original training and develop an adapted curriculum based on staff feedback and operational needs. The first step involved an assessment of the existing training, where BCPH conducted a limited number of informal staff interviews, surveyed the staff of 52 employees, and conducted observations to identify barriers to successful NCCARE360 adoption. The project manager reviewed survey results of open-ended questions and Likert scales, which showed common challenges, including difficulty navigating the platform, lack of clarity on creating a referral workflow (i.e. going from a patient’s profile to referral to a food bank), and insufficient real-world examples in the training materials. The referral rate increased over the review period.

BCPH Health Educators implemented the next phase by developing visual and audio training materials that incorporated step-by-step guides and audio recordings of referral processes. Mock walk-through scenarios were designed to mirror real-life cases handled by BCPH staff, making the training more relevant and engaging. Implementation of these new materials was done in phases, allowing opportunities for staff to provide feedback and suggest refinements. This iterative approach ensured continuous improvement and adaptation. As part of this process, BCPH also recognized that some staff required additional guidance, leading to the introduction of one-on-one training sessions to address specific challenges identified per each program’s needs. For example, the BCPH Women, Infants, and Children program required a specialized document to be uploaded and sent with each referral they received from the BCPH maternity program. This personalized approach ensured all users felt confident navigating the NCCARE360 platform.

## RESULTS

The tailored training program yielded significant improvements in staff engagement and platform utilization. A measurable increase in staff participation in the referral system was observed by the Health Education Supervisor, who acted as the project manager. This increase led to a more efficient connection of clients to community resources.

The project manager reviewed referrals monthly. The number of completed referrals that resulted in successful delivery service increased, demonstrating improved effectiveness of the network. In the first three months after implementation, only a total of 10 referrals were made. In the 10 months after training, the monthly average of referrals made rose to 23.5. Initially five BCPH staff requested additional training opportunities, highlighting the need for ongoing support and refinement. The North Carolina Department of Health and Human Services recognized BCPH’s adapted training as a model for other counties, reinforcing the value of localized customization in statewide initiatives.

## IMPLICATIONS

BCPH’s experience highlights the need to tailor standardized programs to local needs. Flexible training designs ensure state initiatives adapt to workforce capacity, operational limits, and cultural contexts. For example, because the BCPH staff implemented the trainings with program schedules in mind, staff were able to work around the patient schedules. Using iterative improvement frameworks like Plan-Do-Study-Act^7^ keeps training responsive, while involving frontline staff enhances effectiveness. BCPH’s customized training model demonstrates how local adaptations can improve statewide program implementation in resource-limited settings. However, a key limitation is the low participation of community-based organizations, which could be addressed through additional training and onboarding support.

Additionally, this pilot study was limited to one county; a broader multi-county study, particularly in Appalachia, would help assess NCCARE360’s impact across North Carolina’s 100 counties, including the 29 designated as part of the Appalachian Region.^8^ Given that Burke County shares similar health, environmental, and economic demographics with the broader Appalachian Region, this work could benefit other counties by improving NCCARE360 onboarding and training efforts across North Carolina. Expanding participation would create a larger referral network, enhancing service coordination throughout the region. Additionally, organizations serving multiple counties could more effectively reach individuals in need, ensuring greater access to essential resources.
